# Social Anxiety Modulates Default Mode Network Dysfunction and Working Memory Impairment During Social Media‐Like Cue Distraction

**DOI:** 10.1155/da/1506367

**Published:** 2026-11-05

**Authors:** Yuan Hu, Zijie Wei, Lei Xu, Benjamin Becker, Juan Kou

**Affiliations:** ^1^ Institute of Brain and Psychological Sciences Sichuan Normal University Chengdu China sicnu.edu.cn; ^2^ Department of Psychology The University of Hong Kong Hong Kong China hku.hk; ^3^ State Key Laboratory of Brain and Cognitive Sciences The University of Hong Kong Hong Kong China hku.hk

**Keywords:** default mode network, social anxiety, social media distraction, working memory

## Abstract

Recent studies have highlighted the adverse impact of social‐media distractions on cognitive abilities, especially among individuals with anxiety who experience heightened apprehensions about providing immediate online social responses. Nevertheless, the investigation into the mechanism is scarce. In this fMRI study, 94 healthy adults completed a 2‐back working memory (WM) task under three conditions using a between‐subject design: social media‐like cue distraction (e.g., WeChat), nonsocial digital distraction (e.g., browser), and no distraction. Data were analyzed using task‐evoked activation analysis based on the general linear model (GLM) and functional network connectivity (FNC) analysis based on independent component analysis (ICA). Results showed that social media‐like cue distraction impaired WM and altered posterior cingulate cortex (PCC) activity. Crucially, under the social media‐like cue distraction condition, the brain–behavior relationship in the PCC was significantly moderated by trait social anxiety: higher social anxiety predicted a positive coupling between PCC activation and task accuracy, with decreased PCC activation associated with decreased accuracy; conversely, individuals with low social anxiety exhibited decreased PCC activation together with increased accuracy. This study is the first to demonstrate that cues resembling social media can specifically disrupt core brain regions of the default mode network (DMN; PCC) functions during WM tasks and that this neural response is influenced by individual levels of social anxiety.

## 1. Introduction

The rise of social media has significantly altered human social interactions. Despite the apparent benefits of increased social connectivity through digital platforms [1], recent research suggests detrimental impacts on cognitive functions and emotional regulation, which are central to anxiety disorders [2, 3]. Of particular importance is the recognition that excessive use of social media may worsen cognitive weaknesses in individuals with heightened social anxiety, a group known for their sensitivity to social judgment and maladaptive attention patterns [4].

Within these cognitive vulnerabilities, working memory (WM), responsible for temporary information maintenance and manipulation [5], plays a crucial role in linking potential social anxiety to distraction‐related cognitive impairment. WM deficits serve as transdiagnostic markers of psychopathology and exhibit specific associations with social anxiety severity [6]. Importantly, because WM capacity is highly sensitive to internally generated evaluative thoughts and attentional competition, socially salient cues may disproportionately disrupt task performance in socially anxious individuals. Social media notifications, unlike generic social or motivational stimuli, are ingrained in daily digital routines and often convey implicit social evaluation and the urge for an immediate reply. Individuals with elevated social anxiety may prioritize internally evaluating social scenarios prompted by these cues rather than focusing on external task‐related concerns.

Social media’s unique blend of social significance and intermittent reinforcement may disproportionately strain WM resources in individuals with social anxiety. Research suggested that the mere presence of smartphones could diminish WM capacity [7], while neuroimaging studies have associated exposure to social media with increased activity in the default mode network (DMN), particularly in the precuneus, a region involved in self‐evaluation in social contexts [8, 9]. Individuals with social anxiety show increased baseline activation in the DMN when faced with social threats [10], and studies have consistently implicated hyperactivation of the DMN, particularly the posterior cingulate cortex (PCC), in social anxiety and social threat processing. Evidence suggests that socially threatening or evaluative cues abnormally engage PCC/DMN activity, reflecting excessive self‐focused rumination and impaired disengagement from internally generated negative thoughts [11–13]. Furthermore, failure to suppress DMN activity during cognitively demanding tasks has been linked to attentional interference and reduced task performance, particularly under emotionally salient conditions [14, 15].

Accordingly, the present mechanistic hypothesis that social media‐like cues may trigger maladaptive PCC/DMN engagement, may increase attentional control systems (dorsal attention network [DAN]) functional coupling with DMN, and subsequently impair WM performance, especially among individuals with elevated social anxiety (Figure 1). However, there are significant gaps in understanding how the above interacts with social‐media distraction at both behavioral and neural levels.

**Figure 1:**
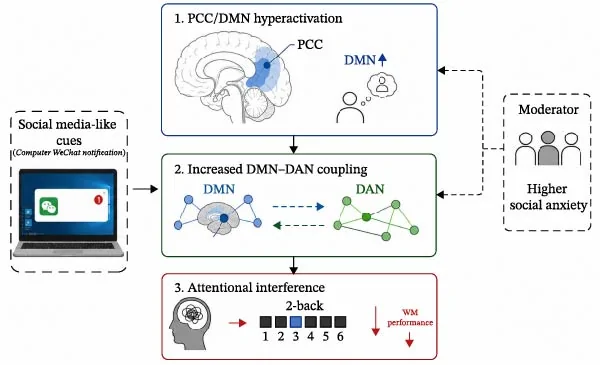
Hypothesis model.

We propose a conceptual model wherein social media‐like cue distraction impairs cognition through a process of competitive resource allocation of the brain (Figure 1). This competition is expected to manifest as altered network activation, disrupted brain activation and internetwork coupling, and consequent behavioral costs.

Specifically, we hypothesize that H1: Behavioral accuracy will be significantly lower in the social media‐like distraction (social‐media distraction [SD]) condition relative to the nonsocial digital distraction (nonsocial distraction [NSD]) and baseline no‐distraction (baseline [BL]) conditions. H2: The SD condition will significantly alter activation in the DMN, particularly in the PCC, compared to the NSD and BL conditions. H3: The SD condition will be associated with significantly stronger functional connectivity between the DMN and the DAN relative to the NSD and BL conditions. H4: Trait social anxiety will significantly moderate the relationship between brain function and behavioral accuracy specifically in the SD condition, such that higher social anxiety predicts a stronger positive coupling between PCC activation and accuracy (i.e., decreased PCC activation associated with decreased accuracy).

The study aims to investigate the differential impact of social and nonsocial media‐like cue distractions on WM performance and the underlying neural mechanisms, particularly focusing on the dynamics between the DMN and the DAN. Additionally, the study seeks to explore how trait social anxiety influences these behavioral and neural responses. By combining social media‐like cue manipulation tasks with functional neuroimaging techniques, the findings aim to enhance the understanding of anxiety‐related cognitive vulnerabilities in the digital era. The results could potentially guide the development of targeted interventions for populations with social anxiety who are disproportionately affected by cognitive demands mediated by technology.

## 2. Methods and Materials

### 2.1. Participants

According to G^∗^Power (Version 3.1.9.7), a sample size of 90 participants (30 per group) was required to achieve 80% power for observing the main effect of distraction condition or related interaction in a two‐way ANOVA with a between‐subject design, based on an effect size of *η* ^2^ = 0.102 [8] and an alpha error value of 0.05 for the investigation of group differences in social‐media distraction on cognition. Ninety‐four right‐handed individuals aged 18–24 (*M* = 19.88, SD = 1.67) participated in the study (41 males and 53 females). Four participants’ fMRI data were excluded due to excessive head movement (>3 mm translation or 3° rotation). The final fMRI analytical sample included 90 participants, with 94 valid behavioral data. All participants were Han Chinese young students recruited from a local university and screened as healthy controls based on self‐report of no current or lifetime history of diagnosed psychiatric or neurological disorders. All participants provided written consent and received financial compensation. The research was approved by the ethics committee of the university. The study was conducted in accordance with the principles of the Declaration of Helsinki.

To control for potential confounding variables, all participants completed validated questionnaires before the experiment. These questionnaires assessed IQ by Raven’s standard progressive matrices (RSPM) [16], depression measured by the Beck depression inventory II (BDI‐II) [17], anxiety evaluated using the state–trait anxiety inventory (STAI) [18], internet addiction assessed by the internet addiction test (IAT) [19], WeChat daily usage duration (e.g., How much time do you typically spend using WeChat each day?), and degree of dependence on WeChat (e.g., To what extent do you agree that WeChat has become an integral aspect of my daily life: 1 – totally disagree; 5 – totally agree). Social anxiety was assessed by the Liebowitz social anxiety scale (LSAS) [20]. As the present study employed a nonclinical sample, LSAS scores were used dimensionally as a continuous measure of trait social anxiety rather than as a diagnostic cutoff. This analytic approach is appropriate for examining individual differences in social anxiety severity within healthy populations.

### 2.2. Study Design and Task

This study employed a between‐subjects design to investigate the effects of different types of online distraction on WM. A between‐subjects design was chosen to avoid carryover effects and demand characteristics that would arise if participants were exposed to multiple distraction conditions, which could reveal the study’s purpose and alter natural responding. Participants were randomly assigned to one of three distraction conditions during a WM task: SD, NSD, or a BL condition with no distraction. The primary task was a 2‐back WM paradigm, administered in both verbal (English letters) and social (face identity) variants. We note that a within‐subjects design, if feasible in future work, could increase statistical power; this is discussed in the Limitations section.

#### 2.2.1. The Verbal and Social WM Task

A two‐back WM task was employed to assess participants’ verbal and social WM according to a previous study [21]. English letters were used as stimuli in the verbal task. Participants were instructed to press the Number 1 key for a match when the displayed letters matched the second pair presented in reverse order and the Number 3 key if there was no match. A set of 24 pairs of distinct capital letters (e.g., AR, AK, AQ, AZ, AJ, RA, RQ, RZ, KA, etc.) was used in the verbal WM task. In the social WM task (based on Xu et al. [22]), participants were presented with facial expressions showing positive, neutral, and negative (angry) emotions as stimuli. In total, 18 faces were used in the experiment, consisting of positive, negative, and neutral expressions. Participants were directed to press the Number 1 key if the displayed faces matched the second pair presented in reverse order and the Number 3 key if there was no match. A block design was used in the study, with each block containing 12 stimuli. The stimuli were displayed on a black background for 2000 ms, and then a fixation point appeared for 500 ms between each stimulus in blocks. One block lasts 29,500 milliseconds. A fixation period, ranging from 11,000 ms, was interposed between blocks. Between blocks, distraction conditions were manipulated between runs, with participants randomly assigned to any of the conditions for one of three runs. Each condition included 12 blocks that lasted for a total of 8 min. The participants had to respond quickly and accurately during the task.

#### 2.2.2. Online Information Distraction Manipulation

The distraction stimuli were designed to mimic the dynamic and multimodal features of actual notifications by capturing a laptop screen showing WeChat messages and modifying the 360 Web Browser download icon popping. During the WM task, task‐irrelevant alerts (social media‐like cue alerts/nonsocial media‐like cue alerts) were presented pseudorandomly. Each alert consisted of a visual pop‐up icon appearing in the peripheral visual field accompanied by its corresponding auditory cue delivered via headphones. The number of alterations of WeChat and the 360 Web Browser was matched between the two conditions.

Critically, to isolate the effect of social components, the SD and NSD conditions were meticulously matched for basic perceptual salience: the icons were of identical size, luminance, and visual complexity (flashing protocol); the auditory cues were matched for approximate duration and intensity; and both shared the same temporal pattern of occurrence. Participants were not explicitly instructed to ignore the distractors; they were simply told to focus on the 2‐back task and respond as accurately and quickly as possible. No reference was made to peripheral alerts in the task instructions.

The manipulation of the three experimental conditions is clearly illustrated in the Video (S1: SD condition, S2: NSD condition, S3: BL condition) and Figure 2. Participants were randomly assigned to one of the three between‐subjects conditions (SD, NSD, and BL). Prior to the formal task, a practice session ensured a minimum performance threshold (≥60% accuracy). Behavioral and neural measures were compared across these conditions to isolate the specific impact of social‐semantic distraction. Visual stimuli were displayed through E‐Prime software (Psychology Software Tools, Pittsburgh, PA), Version 3.0, on a Hewlett–Packard computer that had a screen resolution of 1920 × 1080 and a refresh rate of 60 Hz.

**Figure 2:**
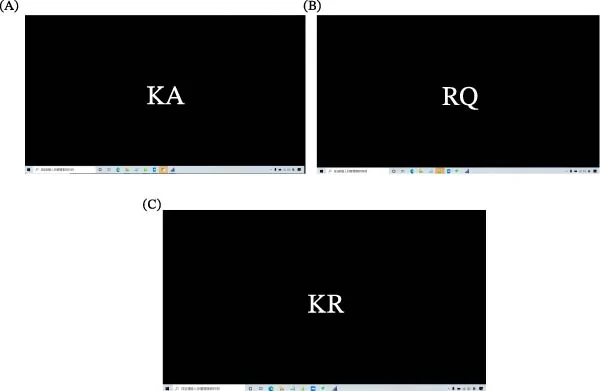
Distraction conditions. Three different distraction manipulation conditions and details of distraction bar (more details in Supporting video). (A) SD (WeChat), (B) NSD (360 Web Browser), and (C) BL.

### 2.3. Behavior Result Analysis

IBM SPSS statistical software (Version 22 Armonk, New York, USA) was utilized to process the behavioral data. An ANOVA with repeated measures was carried out, examining accuracy (ACC) in a 3 (SD, NSD, and BL) × 2 (verbal and social) design. Significant effects were reported along with effect sizes expressed in the partial eta square (*η* ^2^ *p*). Significant interaction effects were examined, followed by appropriate posthoc tests with Bonferroni correction for multiple comparisons.

### 2.4. fMRI Data Collection and Analysis

The neuroimaging data analysis followed a two‐pronged approach: (1) a mass‐univariate, task‐based general linear model (GLM) analysis to identify regional brain activation associated with experimental conditions and (2) a data‐driven independent component analysis (ICA) to derive intrinsic brain networks and examine condition‐specific alterations in functional network connectivity (FNC) between them.

#### 2.4.1. Data Collection

MRI data were collected on a 3T Prisma MR scanner (Siemens, Erlangen, Germany) with a 64‐channel head–neck coil at the university. High‐resolution functional images for task‐based data were acquired using the magnetization‐prepared rapid gradient‐echo sequence (repetition time = 2000 ms, echo time = 30 ms, flip angle = 90°, FOV = 224 mm × 224 mm, number of slices = 62, slice thickness = 2.0 mm, voxel size = 2 mm × 2 mm × 2.3 mm, and multiband factor = 2). T1‐weighted anatomical images were additionally acquired to improve normalization of the functional images (repetition time = 2530 ms, echo time = 2.98 ms, flip angle = 7°, matrix size = 224 × 256, 192 sagittal slices, slice thickness = 1.0 mm, and voxel size = 0.5 mm × 0.5 mm × 1 mm).

#### 2.4.2. Preprocessing

The fMRI data were preprocessed using SPM12 (Welcome Trust Center for Neuroimaging, London, UK). The following preprocessing steps were applied: (1) to achieve magnet‐steady images, the first three volumes of each run were discarded; (2) motion correction of the head using a six‐parameter rigid body algorithm was conducted; (3) tissue segmentation and skull stripping bias‐correction was applied to the high‐resolution structural images; (4) normalization (resampled at 3 mm × 3 mm × 3 mm) to Montreal Neurological Institute (MNI) space; and (5) spatial smoothing with an 8 mm full‐width at half maximum (FWHM) Gaussian kernel was applied. Two participants with excessive head movement (>3 mm translation or >3° rotation) were excluded.

#### 2.4.3. Multiple Comparison Correction

Multiple comparison correction was applied consistently across all analyses. For task‐evoked GLM activation maps, a voxel‐level uncorrected threshold of *p* < 0.001 was combined with cluster‐level false discovery rate (FDR) correction at *p*_FDR_ < 0.05 in SPM12. For the ICA analysis, the optimal number of independent components (ICs) was estimated at 26 using the minimum length description (MDL) method [23]. Prior to ICA‐derived *β* weight and FNC analyses, components were screened using one‐sample *t*‐tests at *p* < 0.001 to ensure task‐relatedness, followed by visual inspection to exclude noise components; four ICs were ultimately retained. For the ICA‐derived *β* weight analysis, no correction for the number of ICs was applied; we will additionally report the corrected results and note that the *β* weight findings do not survive correction for multiple comparisons. For the FNC analysis, our hypothesis specifically concerned the connection between task‐positive (DAN‐like) and task‐negative (DMN‐like) networks; therefore, only the pDMN–DAN connection was tested, and no correction across FNC pairs was applied. One‐way ANOVAs comparing the three distraction conditions were conducted in SPSS for both the *β* weight and FNC analyses, with omnibus tests evaluated at *p* < 0.05. All posthoc pairwise comparisons were Bonferroni‐corrected. The moderation analysis (PROCESS macro v4.0 in SPSS) assessed the interaction term at *p* < 0.05.

#### 2.4.4. GLM Modeling for Brain Univariate Activation Effects of Task‐Evoked Regional Signal Amplitude

A two‐level random‐effect GLM analysis was conducted. First level GLMs for the fMRI data included separate regressors for the verbal and social (face memory) WM within SD, NSD, and BL conditions. In order to improve motion control, six motion parameters were included as covariates. The calculated contrasts were then analyzed in a second‐level random‐effects analysis. A high‐pass filter of 128 s was applied to remove low‐frequency drifts. On the group level, the following analyses were conducted: (1) a 2‐way interaction effect was analyzed using ANOVA, with the distraction condition (SD, NSD, and BL) as the between‐factor and stimuli type (verbal and social) as the within‐factors in a flexible factorial design; (2) compare three distraction conditions across all trials using one‐way between‐subjects ANOVA in SPM12 to determine the main effect of the distraction condition; and (3) posthoc paired *t* contrasts were performed if significant interactions or main effects were present. The significance comparison level was set at *p* < 0.05 (two‐sided) with peak‐level FDR correction and the cluster‐defining voxel threshold at the default of 0.001 according to a previous study at the whole brain level [24].

#### 2.4.5. Group ICA (GICA) Analysis

ICA is a technique widely utilized for data‐driven source separation, breaking signals down into individual components [25]. Following preprocessing, the fMRI data was analyzed utilizing the GICA of fMRI Toolbox (GIFT, v4.0c; https://trendscenter.org/software/) in order to decode spatially independent and temporally correlated networks. The ICA analysis in GIFT was conducted in three steps. Initially, principal component analysis (PCA) was utilized to decrease the data dimension for each participant [26]. Furthermore, ICA was implemented to identify the underlying components present in the data. The MDL method [23] was utilized to estimate the number of components for each subject, a widely used technique in the field. To ensure the reliability of the ICA components, the ICASSO3 toolbox in GIFT was employed. This toolbox performs multiple iterations of ICA on the data and calculates a stability index (iq) for each component [27]. In order to guarantee that conclusions were not influenced by randomness or background noise, the ICASSO3 analysis was repeated 100 times to obtain a precise assessment of each component’s reliability in the sample across iterations. In addition, since the ICs produced by ICA were group components, it was necessary to conduct back‐reconstruction using the GICA algorithm. This required using the combined ICA components and the earlier results of PCA to compute individual subject components. Spatial maps and time courses derived from the back‐reconstruction for each subject capture the individual differences manifested by the ICA‐derived components. After reconstructing the spatial maps and converting them into *Z* values for each subject, all components were visually inspected. Only nonartifactual components were chosen.

The GIFT temporal sorting method was used to calculate the degree of task‐relatedness of the components by sorting them based on their time courses, in line with the previous findings. This method enabled the identification of components that show temporal correlation with particular task conditions [8]. Temporal sorting was similar to GLM regression on fMRI voxel time courses, but regression was performed on subject‐level ICA time courses [28]. The resulting *β* weights represent the degree of involvement of the task event‐related component network [8, 29]. In order to improve motion control, six motion parameters were included as covariates. Following the temporal sorting analysis, one‐sample *t*‐tests were conducted on the *β* values of each component, and components with *p* < 0.001 were subsequently selected.

Finally, out of the 26 ICs estimated as optimal by the MDL method, four effective components were identified belonging to three networks: the posterior DMN (pDMN: IC 17), the DAN (IC 3), and the high visual network (VN: ICs 4, 7). Identification of the ICs was performed by visually examining the spatial overlap with canonical network templates and assigning labels based on the component label provided by GIFT (i.e., selecting the network with the highest spatial correlation to the established templates). A one‐way ANOVA was conducted in SPSS to compare the selected components of participants under different task conditions (*β* values of temporal sorting analysis), assessing the main effects of the distraction conditions (SD, BL, and NSD) [28].

#### 2.4.6. FNC (Temporal Cofluctuation Between Networks)

To test our specific hypothesis regarding the effect of social distraction on internetwork coupling, we analyzed the FNC between the spatially and temporally filtered components identified in the previous step. Our analysis was hypothesis‐driven, focusing explicitly on the connection between the pDMN and the DAN. The strength of this predefined pDMN–DAN connection was extracted and compared across the three distraction conditions (SD, NSD, and BL) using the multiple analysis of covariance (MANCOVA) module in the GIFT toolbox. A band‐pass filter with a filtering range of 0.017–0.15 Hz was used to denoise the time course of each IC [30], and the detrend (linear, quadratic, and cubic trends), despike, and regression head movement parameters were processed [31]. The FNC domain matrix was then derived through calculating the Pearson correlation coefficient between the average time series of each chosen pair of ICs. This FNC domain matrix was converted by Fisher’s *Z*‐transformation and then extracted. Following the extraction of the coefficients of the FNC domain matrix, a one‐way ANOVA was performed in SPSS to analyze the main effects of the distraction conditions (SD, BL, and NSD) and compare participants’ FNC domain matrix coefficients across various task conditions.

### 2.5. Associations Between Social Anxiety Degree/Behavioral and Brain Responses to Social Information Distraction

Pearson correlation analysis was used to examine associations between significant group differences in behavioral and neural responses. To test our hypothesis that trait social anxiety moderates the relationship between neural response and behavioral performance specifically under social distraction, we conducted a moderation analysis. This analysis focused exclusively on the SD condition. Using the PROCESS macro (v4.0) for SPSS, we estimated a regression model with behavioral accuracy during the SD condition as the outcome variable. The model included the centered neural predictor: the activation parameter estimate (*β*) from the PCC cluster that showed a significant main effect of the SD condition (Results section). The moderator: the continuous score from the LSAS. The interaction term: the product of the centered PCC activation and the centered LSAS score. A significant interaction term would indicate a moderation effect. To probe and visualize a significant interaction, we utilized the Johnson–Neyman technique as implemented in PROCESS, which identifies the range of moderator values (LSAS scores) for which the relationship between PCC activation and behavioral accuracy is statistically significant. All reported effects are based on a significance threshold of *p* < 0.05. To isolate the unique effect of social anxiety from general anxiety and depressive symptoms, we included the STAI and BDI‐II total scores as covariates in the moderation model. The model thus tested the interaction between PCC activation and the LSAS‐anxiety score on behavioral accuracy during the SD condition while controlling for STAI and BDI‐II.

## 3. Results

### 3.1. Self‐Reported Measures

Results showed no statistically significant differences in age, gender, STAI, BDI, IQ, LSAS, and WeChat daily use time variables among the three experimental groups (Table 1). Additionally, the internal consistency reliability for the LSAS scale (Cronbach’s *ɑ* = 0.865), the LSAS‐anxiety subscale (Cronbach’s *ɑ* = 0.758), and the LSAS‐avoid subscale (Cronbach’s *ɑ* = 0.753) was found to be acceptable within our sample.

**Table 1:** Characteristics of the participants.

Characteristic	*SD*	*BL*	*NSD*	*χ^2^/F*(*p*)
*N* (M/F)	32 (14/18)	32 (15/17)	30 (12/18)	0.30 (0.86)
Age	19.75 (1.88)	20.38 (1.85)	20.33 (1.52)	1.25 (0.29)
RSPM	48.88 (6.52)	49.13 (5.74)	49.17 (6.05)	0.02 (0.98)
BDI	7.16 (10.22)	9.34 (9.41)	9.40 (7.01)	0.64 (0.53)
SAI	38.41 (11.91)	41.16 (9.67)	43.20 (9.43)	1.66 (0.20)
TAI	42.06 (10.39)	45.34 (9.92)	44.97 (7.35)	1.17 (0.32)
IAT	52.75 (13.36)	53.84 (6.52)	48.80 (11.96)	1.31 (0.27)
LSAS‐anxious	29.34 (15.21)	30.47 (15.48)	30.93 (11.70)	0.10 (0.90)
LSAS‐avoidance	25.72 (14.53)	26.84 (14.15)	28.80 (10.95)	0.42 (0.66)
WeChat daily use time	3.33 (1.31)	3.88 (1.50)	4.03 (1.27)	0.60 (0.55)
WeChat dependence	4.22 (0.87)	4.00 (0.88)	4.07 (1.02)	0.47 (0.63)
Handedness	Right‐handed (32)	Right‐handed (32)	Right‐handed (30)	0 (1)
Ethnicity	Han Chinese ethnicity (32)	Han Chinese ethnicity (32)	Han Chinese ethnicity (30)	0 (1)

### 3.2. Behavioral Group Difference

The results, based on data from 94 participants with valid behavioral responses, indicated a significant main effect of distraction condition (*F*_(2,91)_ = 3.249, *p* = 0.043, *η* ^2^ *p* = 0.067) with lower accuracy during social‐media information distraction conditions (SD < BL, *p*_Bonferroni_ = 0.037, *d* = 0.606) but not in comparison between SD and NSD (*p*_Bonferroni_ = 0.598), NSD and BL (*p*_Bonferroni_ = 0.683), and a significant main effect of stimuli (*F*_(2,91)_ = 9.315, *p*_Bonferroni_ = 0.003, *η* ^2^ *p* = 0.093) with higher accuracy during verbal WM than social memory task. The lack of a significant two‐way interaction (*F*_(2,91)_ = 3.45, *p* = 0.684, *η* ^2^ *p* = 0.008) reflects that both the verbal and social conditions were affected by social‐media distraction **(**Figure 3A). Descriptive statistics across the three distraction conditions (Table S1), as well as the corresponding main effects with sex included as a covariate, are provided in Supporting Information.

**Figure 3:**
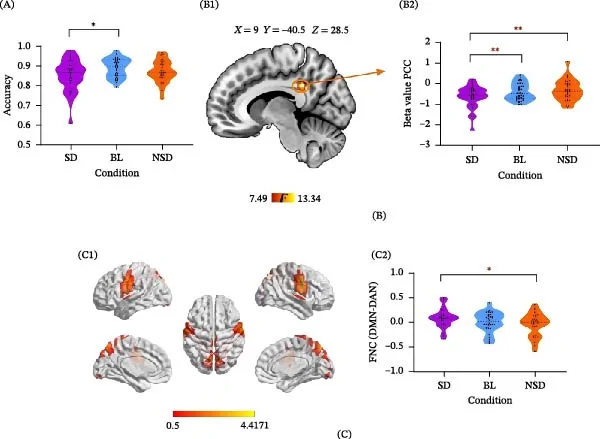
Behavioral and neural group differences. (A) Decreased working memory behavioral accuracy in the social media‐distraction condition; (B1) whole brain peak activation map of main effect of condition (*p*_FDR_peak = 0.042); (B2) posthoc group comparisons analysis of main effect in the PCC; (C1) main effect of condition for functional connectivity in the pDMN–DAN (*p* = 0.043); (C2) posthoc group comparisons showed significant increased pDMN–DAN functional connectivity in the social media‐distraction condition  ^∗^ *p* < 0.05,  ^∗∗^ *p* < 0.01.

### 3.3. Brain Activiation Results

#### 3.3.1. Comparisons Between Different Conditions on Neural Activation

In line with the behavioral results, no significant condition × stimuli type interaction effect was observed, but the results revealed a significant main effect of the distraction condition located in the right posterior cingulate gyrus (PCC) extending to the right precuneus according to the AAL3 template [32] (peak MNI, *xyz* = [12, −40, 30], *F*_(2,87)_ = 13.34, *p*_*FDR*_ peak = 0.042, cluster size = 24), posthoc comparisons revealed lower activation in the SD condition when compared to the other two conditions (*ps*_Bonferroni_ < 0.001), indicating that social information distraction may induce stronger suppression of the PCC/precuneus than control and nonsocial conditions **(**Figure 3B).

#### 3.3.2. Comparisons Between Different Conditions of ICA and Neural Function Connectivity (pDMN–DAN)

Four effective ICs were selected from 26 ICs, which involved three networks: high VN (ICs 4, 7), DAN (ICs 3), and pDMN (ICs 17). There was a significant main effect of HVN (IC7) involvement between distraction conditions (*F*_(2,87)_ = 3.344, *p* = 0.040, *η* ^2^ *p* = 0.073), showing that SD > BL (*p*_Bonferroni_ = 0.038, *d* = 0.589), not in other comparison between SD and NSD (*p*_Bonferroni_ = 0.274) and NSD and BL (*p*_Bonferroni_ = 1.000). However, this effect did not survive Bonferroni correction for the number of ICs tested (four ICs; Bonferroni‐corrected *p* = 0.160). No other IC *β* weights showed significant condition effects. Therefore, the *β* weight results should be interpreted with caution.

There was a significant main effect of FNC strength of pDMN–DAN between distraction conditions (*F*_(2,87)_ = 3.278, *p* = 0.043, *η* ^2^ *p* = 0.072), showing that SD > NSD (*p*_*Bonferroni*_ = 0.041, *d* = 0.569) not in comparisons between SD and BL (*p*_*Bonferroni*_ = 0.278) and NSD and BL (*p*_*Bonferroni*_ = 1.000) (Figure 3C). Supplementary analyses with sex included as a covariate confirmed the robustness of the main effect (IC7; pDMN–DAN), which remained significant (see the Supporting Information for full details).

### 3.4. Associations Between Altered Brain Function and Behavioral Responses With the Level of Social Anxiety

We further evaluated whether the interaction between social anxiety and the neural activation of PCC can predict the behavioral accuracy increases during the SD condition utilizing modulation analysis in SPSS 22. A significant modulation effect was found, suggesting that the LSAS‐anxiety levels significantly moderate the association between levels of neural activation of PCC and behavioral accuracy responses during the SD condition (*B* = 0.616, *SE* = 0.234, *t* = 2.636, *p* = 0.015, Δ*R* ^2^ = 0.222, Cohen’s *f* ^2^ = 0.316). Further disentanglement of the effect using the Johnson–Neyman technique [33] revealed that, when LSAS‐anxiety was below 25.2 in the sample distribution, the level of PCC activation during the SD condition negatively influenced behavioral accuracy in the 2‐back task. Conversely, when LSAS‐anxious scores were above 52.3, the level of PCC activation during the SD condition positively influenced behavioral accuracy in the 2‐back task (Figure 4). Between these two values (25.2 and 52.3), the relationship between PCC deactivation and accuracy was not statistically significant. In plain terms, these results indicate that the nature of the brain–behavior relationship shifted depending on social anxiety severity: for individuals with relatively low social anxiety, stronger PCC deactivation during social distraction was linked to better task performance, whereas for those with relatively high social anxiety, stronger PCC deactivation was linked to poorer performance.

**Figure 4:**
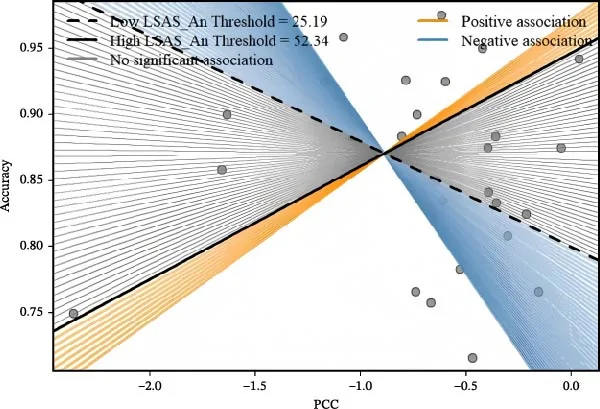
Modulation relationship. The modulation role of social anxiety between PCC activation and behavioral accuracy in SD condition.

To confirm that the moderating effect of social anxiety is specific to the social media‐like distraction condition, the same moderation model was tested in the NSD and BL conditions. In the NSD condition, the interaction between PCC activation and LSAS‐anxiety scores was not significant (*B* = 0.151, SE = 0.141, *t* = 1.073, *p* = 0.293; Δ*R* ^2^ = 0.038, Cohen’s *f* ^2^ = 0.044). Similarly, no significant moderation effect was observed in the BL condition (*B* = −0.045, SE = 0.208, *t* = −0.217, *p* = 0.830; Δ*R* ^2^ = 0.002, Cohen’s *f* ^2^ = 0.002). These null findings confirm that the moderating role of social anxiety on the PCC–accuracy relationship is specific to the social media‐like distraction condition.

To address the potential influence of general anxiety and depressive symptoms, we conducted a supplementary analysis that included the STAI and BDI‐II scores as covariates. The critical interaction effect remained significant in this model (standardized *B* = 0.527, *SE* = 0.237, *t* = 2.223, *p* = 0.037, Δ*R* ^2^ = 0.163, Cohen’s *f* ^2^ = 0.225), with the Johnson–Neyman region boundary shifting slightly to 23.1. The persistence of the significant moderation effect across both analytical approaches underscores the robustness of the finding that trait social anxiety qualitatively shapes the PCC–accuracy relationship during social distraction.

To characterize the minimum detectable effect size for the moderation model given the present sample size, a posthoc sensitivity analysis was conducted in G^∗^Power (Version 3.1.9.7). Using a linear multiple regression framework with *ɑ* = 0.05 (two‐tailed), 80% power, one tested predictor (the PCC × LSAS interaction term), and three total predictors, the analysis indicated that the study was sensitive enough to detect an interaction effect of *f^2^* ≥ 0.282. The observed interaction effect in the primary moderation model (*f^2^* = 0.316) exceeded this threshold, confirming adequate sensitivity. With the inclusion of additional covariates (STAI and BDI‐II; five total predictors), the minimum detectable effect size was *f^2^* ≥ 0.284. The observed effect in this model (*f^2^* = 0.225) fell slightly below this threshold, suggesting that the moderated effect, while still statistically significant, may have been modestly attenuated after controlling for general anxiety and depressive symptoms. We therefore interpret the supplementary model as providing convergent but conservative evidence, and we note that future studies with larger samples would be beneficial to more precisely estimate the moderation effect when multiple covariates are included.

## 4. Discussion

This study aimed to investigate the differential impact of distractions via online social media‐like cues (mimic social‐media distraction) on verbal and social WM performance, as well as the underlying neural mechanisms. The findings revealed that distractions from social media‐like cues were associated with decreased behavioral accuracy in both verbal and social WM tasks, whereas distractions from nonsocial media‐like cues (e.g., web browser downloading alerts) did not. This indicates a significant impact of online social media‐like cues on WM performance at the behavioral level within the experimental context. At the neural level, distractions from online social information were linked to reduced activation in the right PCC/precuneus, a key brain region within the pDMN, and increased functional coupling between the pDMN and DAN. Furthermore, the degree of social anxiety influenced the link between PCC activation and behavioral accuracy when exposed to distractions from online social media‐like cues.

### 4.1. Social Media‐Like Cue Distraction Reduced WM Behavioral Performance Accuracy

According to the current results, the distraction caused by social media‐like cues has a significant effect on WM performance, which partly confirms previous studies [34]. WM and other executive functions critically rely on efficiently controlling distractions [35]. A reduced ability to resist distraction has been proposed as a potential factor contributing to declines in WM, leading to cognitive impairment deficits [35]. Prior studies have demonstrated that unexpected social‐media notifications can interrupt focus and result in a decrease in global and local attention while engaging in external activities [8, 36].

Besides the findings noted by Hadar et al. [34], it has been demonstrated that the mere proximity of an individual’s smartphone can hinder performance on a WM task [7]. Even when the device was turned off, participants exhibited consistent outcomes. However, their performance notably improved when their phone was in a nearby room compared to situations where it was stored in their bag or placed on the desk (muted and facing downward). When you mute or put your phone downward, you can reduce online distractions, which gives proof of our study’s manipulation backwards. There was a significant group difference between social media‐like cues and the BL; however, no significant difference was found between the social media‐distraction condition and the nonsocial media‐distraction condition. This suggests that the distraction effect of social media may not be significantly stronger than that of nonsocial media. It could also be because we tried to mimic social‐media distraction as accurately as possible, but this was still a laboratory condition. Future studies should incorporate real interactions. Furthermore, while a practice accuracy criterion (over 60%) was used to ensure task understanding, future studies might employ a higher threshold to further guarantee a robust baseline of WM performance prior to testing.

### 4.2. Social Media‐Like Cue Distraction Alters PCC Activation and Temporal Coupling Between the DMN and DAN in WM Processing

The results of the present study indicate that, when contrasted with the BL and NSD conditions, there is a significant increased task‐related suppression in the PCC during SD conditions. The PCC is a key component of the DMN [37], supporting internally directed cognition like daydreaming, self‐referential thought, autobiographical memory retrieval, and future planning. While the PCC/precuneus is commonly suppressed during external task execution [37–39], previous studies have also linked this region to external attention and maintaining task performance in the context of negative social evaluation [9, 21]. The social media‐like distraction may thus be associated with a greater cognitive load on WM tasks, leading to a heightened need for deactivation of DMN regions associated with internal attention or disengagement from self‐referential information. Previous studies have shown that as the WM load increases, the extent of DMN deactivation also intensifies [40–44]. Consequently, in the presence of heightened cognitive load linked with social information intrusion, the brain might suppress the PCC to allocate extracognitive resources for managing this interference.

Moreover, under SD conditions, there was a significant increase in functional connectivity between the pDMN and the DAN when compared to NSD conditions. During externally directed attention, the DMN is prominently deactivated, while the DAN is primarily associated with task‐positive functions and top‐down attention control [45]. Thus, the DMN and DAN are generally thought to have a competitive dynamic [46, 47].

Nonetheless, a prior study has pointed out that this competitive dynamic is not permanent. In certain situations, these networks might show positive correlations, especially during mind‐wandering [48–50], memory recall [51], social WM [52], and WM unrelated to immediate perceptual input [53]. Fornito et al. [51] reported that improved task performance was correlated with the dynamic interaction between the DMN and task‐positive networks, a pattern observed in the subgroup with lower dependence scores in the current study. Additionally, the DMN might not exclusively pertain to internally directed attention; the pDMN might also be involved in balancing internal and external focus [54]. This suggests that individuals engaged in external tasks may need to increase the positive coupling between the pDMN and DAN in response to social information intrusion to manage the automatic shift of internal attention and meet the demands of external tasks.

Univariate activation and functional connectivity highlight different aspects of neural dynamics. A decrease in DMN suppression during distraction does not necessarily mean more internally focused processing but rather could indicate a failure to fully disengage from internally generated thoughts. Moreover, the heightened coupling between DMN and DAN may suggest increased interaction between internally and externally focused systems, possibly due to a competition for cognitive resources. Therefore, the current findings may not solely indicate heightened DMN engagement but rather suggest a scenario of reduced network segregation and increased interference between different networks during social‐media distraction.

### 4.3. Social Anxiety Degree Modules the Social Media‐Like Cue Distraction on PCC Activation and WM Behavioral Processing

Underscoring previous results on the impact of social anxiety on digital addiction‐related brain changes [55], variations in the level of social anxiety influenced the relationship between PCC engagement and behavioral accuracy during social‐media information distraction. The low social anxiety subgroups (less than 25 in LSAS‐anxious participants) showed a negative correlation between behavioral accuracy and PCC activation during social‐media information alteration. This suggests that by inhibiting their DMN‐related brain regions, they can improve their WM task performance, which is consistent with previous study [52, 53, 56]. The high social anxiety subgroups (over 52) showed a positive relationship between behavioral accuracy and PCC activation during social‐media information alteration. This suggests that despite inhibiting the PCC gyrus, they were unable to achieve high accuracy scores, possibly reflecting a higher vulnerability of individuals with higher social anxiety for social media‐related distractions. Social media’s constant availability influences individuals’ expectations and desires for social connection, as seen in studies by Marino et al. [57] and Nesi et al. [58]. Individuals anticipate that their peers could be constantly reachable through social media and would promptly respond to their posts or interactions online, as noted by van Driel [59]. Individuals who are unable to respond promptly on social media may face negative social judgment, especially if they have high social anxiety traits. As a result, they may become more vigilant about social‐media information, potentially leading to increased anxiety and a decrease in overall quality of life in the long term. Having access to prompt and appropriate responses is crucial for effectively managing social anxiety and maintaining healthy relationships but can also be accompanied by cognitive distraction as well.

Furthermore, the coupling between PCC and behavior was specifically observed under salient audiovisual distraction in social anxiety‐dependent conditions, not in nonsocial anxiety‐dependent conditions. This finding suggests that attentional capture or arousal cannot solely explain this phenomenon. Social media‐like cues, compared to browser‐like notifications, may have a stronger impact on individuals with high social anxiety due to their interpersonal and self‐evaluative significance. This preference could lead to the engagement of PCC‐related self‐referential processes during WM tasks (via [13]). However, the possibility of broader motivational salience mechanisms should not be disregarded and warrants further investigation through physiological arousal measures and better‐matched control cues in future studies.

Our findings may be helpful for understanding daily social‐media behavior and cognitive function. However, as this study employed a cross‐sectional, within‐subject design, causal interpretations (e.g., that social‐media distraction causes cognitive impairments in socially anxious individuals) cannot be made. Future longitudinal or intervention studies (e.g., employing distraction‐blocking apps or cognitive training) are necessary steps to establish causality and to explore how these laboratory‐observed effects translate to real‐world academic performance and clinical functioning.

In summary, these results provide new insights into understanding cognitive task performance and its neural underpinnings under more naturalistic conditions of information intrusion, further emphasizing the importance of moderate control over social‐media use in maintaining cognitive function.

## 5. Conclusion

The study investigated the impact of social media‐like cue information distraction on WM in youths. It was found that social‐media distractions were associated with impaired performance in both verbal and social WM. Neural findings showed decreased activation in the PCC and increased functional temporal coupling between the pDMN and DAN. Additionally, the level of social anxiety was found to modulate the relationship between brain function and behavioral response accuracy. This suggests that social anxiety should be considered in interventions and research on the effects of social‐media distraction in the future.

## 6. Limitation

The study has limitations that should be acknowledged. First, the social media‐distraction condition aimed to simulate common social‐media notifications using brief icons and sound alerts (WeChat) instead of real‐time social‐media feeds. Therefore, the findings reflect neural responses to social media‐like cues at the WM level, not actual engagement with the use of social media. Furthermore, WeChat differs from other social‐media platforms and cultures, potentially causing an overrepresentation in the current study and restricting its generalizability. Second, social‐media notifications differ from generic social or motivational stimuli not in their content but in their associations with habitual checking, implicit social judgment, and intermittent reinforcement. While the study partially captures these cue‐level characteristics, it does not replicate the dynamics of scrolling, content evaluation, or social interaction feedback explicitly (include both positive and negative social feedback). Future research using more realistic paradigms, like simulated social‐media feeds or naturalistic interaction tasks, could improve the findings. Thirdly, the assessment of social‐media use was based on self‐reported WeChat‐specific items and did not systematically survey multiple social‐media platforms. A specific measure of habitual notification‐checking frequency (the cue‐driven behavior most directly relevant to the distraction paradigm employed) was not included. Future research would benefit from standardized multiplatform instruments that capture primary platforms, daily usage duration, and notification‐checking habits to enable more comprehensive measurement and facilitate cross‐study comparisons. Fourth, the between‐subjects distraction design, while chosen to avoid carryover effects and demand characteristics, limited statistical power compared to a within‐subjects approach. Future studies could consider within‐subject manipulation of distraction conditions to enhance statistical power.

Lastly, the study sample included healthy young adults aged 18–24 who were all Han Chinese students recruited from a single university, which limits the generalizability of the findings across ethnic, cultural, and educational backgrounds. In one group, there were around 30 participants, with a limited sample size to explain the modulation effect of social anxiety. Replication in culturally and demographically diverse samples is needed to assess the generalizability of the observed neural mechanisms. It may restrict the applicability of the findings to different age groups or clinical populations. Considering the diverse patterns of social‐media usage among various age groups and contexts, future research should explore if comparable neural mechanisms are involved in adolescents or individuals with social anxiety disorder to understand the current modulation effect of the social anxiety trait better. Current researchers used only the self‐report LSAS‐ anxiety subscale to measure social anxiety. To improve the trustworthiness and coherence of outcomes, upcoming studies should consider employing multiple assessment approaches, like clinician ratings (such as DSM‐5).

## Funding

This study was supported by the STI 2030‐Major Projects (Grant 2022ZD0210900), the National Nature Science Foundation of China (Grant NSFC32100893), the Foundation of Sichuan Research Center of Applied Psychology of Chengdu Medical College (Grant CSXL‐23308), and the Ministry of Education Foundation on Humanities and Social Sciences (Grant 24YJC190036).

## Disclosure

After using the tool/service (Stork), the authors then revised and edited the content as required, and bear full responsibility for the publication’s content. The sponsors played no role in the study design, data collection, analysis, or interpretation, writing the report, or the decision to submit the article for publication.

## Ethics Statement

The study was conducted in accordance with the principles of the Declaration of Helsinki. It was approved by the local Ethics Committee. Written informed consents were provided by participants (Number 2023101).

## Conflicts of Interest

The authors declare no conflicts of interest.

## Supporting Information

Additional supporting information can be found online in the Supporting Information section.

## Supporting information


**Supporting Information** This section contains the following Supporting Information that provide visual documentation of the experimental manipulations and additional statistical results: SD condition.mp4: A video recording demonstrating the experimental procedure under the social‐media distraction (SD) condition. This video illustrates the presentation of task‐irrelevant social alerts, featuring a laptop screen capture showing WeChat message pop‐up icons with synchronized auditory cues, pseudorandomly presented during the working memory task. NSD condition.mp4: A video recording demonstrating the experimental procedure under the nonsocial distraction (NSD) condition. As described in Section 2.2.2, this video shows the presentation of task‐irrelevant alerts consisting of a visual pop‐up icon (modified 360 Web Browser download icon) appearing in the peripheral visual field, accompanied by a corresponding auditory cue delivered via headphones, while participants concurrently perform the 2‐back task. BL condition.mp4: A video recording demonstrating the full experimental procedure under the baseline (BL) condition, in which participants performed the verbal and social 2‐back working memory task without any peripheral distraction alerts. Table S1. Behavioral accuracy and reaction time across the three distraction conditions.

## Data Availability

The data that support the findings of this study are available upon request from the corresponding author. The data are not publicly available due to ethical restrictions.
