# Supplementary material for: Social Anxiety Modulates Default Mode Network Dysfunction and Working Memory Impairment During Social Media‐Like Cue Distraction
Source: Depress Anxiety. 2026 Nov 5;2026:1506367. doi: 10.1155/da/1506367 (PMC13740678; doi:10.1155/da/1506367)
Supplement: Supplementary file 1 [file DA-2026-1506367-s001.zip › Supplementary Information.docx]

**Supplementary Information for**

**“Social Anxiety Modulates Default Mode Network Dysfunction and Working Memory Impairment During Social Media-like Cues Distraction”**

**Supplementary Results**

Sex has been calculated as a covariate in this section to be transparent.

**Behavioral results:** The main effect of distraction condition remained significant (*F_(2,89)_* = 3.207, *p* = 0.045, η²*p* = 0.067; SD < BL, *p_Bonferroni_* = 0.040; BL > NSD, *p_Bonferroni_* = 0.784; NSD > SD, *p_Bonferroni_* = 0.536).

****Brain results:****

**HVN (IC7) β weight:** The nominal effect remained (*F_(2,86)_* = 3.313, *p* = 0.041, η²*p* = 0.073; SD > BL, *p_Bonferroni_* = 0.039; SD > NSD, *p_Bonferroni_* = 0.285; NSD > BL, *p_Bonferroni_* = 1), though this did not survive correction for the number of ICs tested.

**FNC (pDMN-DAN):** The main effect remained significant (*F_(2,86)_* = 3.294, *p* = 0.042, η²*p* = 0.073; SD > NSD, *p_Bonferroni_* = 0.040; SD > BL, *p_Bonferroni_* = 0.287; NSD > BL, *p_Bonferroni_* = 1).

**Moderation (primary model):** The interaction remained significant (B = 0.629, SE = 0.239, *t* = 2.632, *p* = 0.016, ΔR² = 0.229, Cohen's f² = 0.330).

**Moderation (with BDI/STAI covariates):** The interaction remained significant (B = 0.537, SE = 0.243, *t* = 2.205, *p* = 0.039, ΔR² = 0.167, Cohen's f² = 0.232).

****Table S1.**** Behavioral accuracy and reaction time across the three distraction conditions.

| Measure | Condition | n | M | SD | *F(2, 91)* | *p* | η²*p* |
| --- | --- | --- | --- | --- | --- | --- | --- |
| ****Accuracy**** | SD | 32 | 0.850 | 0.079 | 3.249 | .043 | .067 |
|  | BL | 32 | 0.889 | 0.047 |  |  |  |
|  | NSD | 30 | 0.870 | 0.054 |  |  |  |
| ****RT (ms)**** | SD | 32 | 1033.83 | 163.14 | 1.013 | .367 | .022 |
|  | BL | 32 | 1000.03 | 148.64 |  |  |  |
|  | NSD | 30 | 981.35 | 127.66 |  |  |  |

Note. SD = Social media-like Distraction; NSD = Non-social Digital Distraction; BL = Baseline (no distraction). RT = reaction time in milliseconds. Post-hoc comparisons for accuracy: SD < BL, *p_Bonferroni_* = 0.037; SD vs. NSD and NSD vs. BL were not significant (*ps* > 0.05).
